# Detection of SARS-CoV-2 and a possible variant in shelter cats

**DOI:** 10.1371/journal.pone.0317104

**Published:** 2025-01-13

**Authors:** Ogi Okwumabua, Nancy Bradley-Siemens, Catherine Cruz, Lauren Chittick, Melissa Thompson

**Affiliations:** 1 Department of Pathology and Population Medicine, College of Veterinary Medicine, Midwestern University, Glendale, Arizona, United States of America; 2 Department of Small Animal Shelter and Community Medicine, College of Veterinary Medicine, Midwestern University, Glendale, Arizona, United States of America; 3 Arizona Humane Society, Phoenix, Arizona, United States of America; Chulalongkorn University Faculty of Medicine and King Chulalongkorn Memorial Hospital, THAILAND

## Abstract

SARS-CoV-2 is the cause of mild to severe acute respiratory disease that led to significant loss of human lives worldwide between 2019 and 2022. The virus has been detected in various animals including cats and dogs making it a major public health concern and a One Health issue. In this study, conjunctival and pharyngeal swabs (n = 350) and serum samples (n = 350) were collected between July and December 2020 from cats that were housed in an animal shelter and tested for the infection of SARS-CoV-2 using real time reverse-transcription polymerase chain reaction (rRT-PCR) that targeted the N1 and N2 genes, and a SARS-CoV-2 surrogate virus neutralization Test (sVNT), respectively. 203 (58%) swab samples were negative (N1 and N2 not detected), 2 (0.6%) were positive (N1 and N2 detected) and 145 (41%) were inconclusive (only N1 detected). Analysis of the N2 region and multiple sequence alignment revealed base-pair deletions and substitutions in the N2 probe binding region of the feline samples RNA extracts in comparison with the positive control and human SARS-CoV-2 sequences in the GenBank database. Substituting the N2 probe with a probe derived from the cat sample amplicon sequences, 123 of 127 (96.9%) of the N2 negative samples returned positive. All but one of the 350 serum samples were negative for SARS-CoV-2 antibody. These observations indicated that although detection of SARS-CoV-2 infection was low in the samples tested, pet cats can harbor the virus and serve as potential source for virus spread that may lead to human infections. Additionally, cats may harbor a yet-to-be described virus that is somewhat related to SARS-CoV-2.

## Introduction

SARS-CoV-2 causes severe respiratory diseases in humans. Its infections lead to over 584 million confirmed human cases, with more than 6.4 million deaths worldwide between 2019 and 2022. Cases of SARS-CoV-2 have been reported in companion (cats and dogs), wild, and domestic animals [1,2]. Transmission from infected owners to cats and dogs have also been reported [3,4] indicating its significance in One Health medicine.

Since the emergence of SARS-CoV-2 several variants have emerged [5]. The biological and clinical significance of emerging SARS-CoV-2 variants makes it worthwhile to continue monitoring human and animal populations for exposure to the virus to gain insight on evolution of variants, virus spread, vaccine and diagnostic reagent development.

In the United States and presumably worldwide, dogs and cats are the most common animal species taken as pets. In the United States alone, 26% of households have cats estimated at 60–62 million cats [6]. Both species (cats and dogs) are reportedly naturally and experimentally susceptible to SARS-CoV-2 with cats exhibiting higher susceptibility than dogs [7–9]. Despite efforts to control COVID-19, the virus is still circulating amongst human and animal populations.

The cats in this study were surrendered to the shelter by their former owners or brought to the shelter as strays from the community. Animal shelters provide care and protection for animals, help reunite lost pets with their owners, and find new homes for animals that are lost, abandoned, or surrendered to shelters by owners. This makes shelters conducive to conduct studies on SARS-CoV-2 by using samples obtained from the shelter cats from naturally infected animals from diverse households and varied locations in a managed intake environment to evaluate exposure incidence of the virus while contributing knowledge to public health. During the SARS-CoV-2 pandemic a reverse transcription real-time polymerase chain reaction (rRT-PCR) assay that targeted two regions within the virus nucleocapsid (N) gene, designated N1 and N2, respectively, was developed, validated and widely used for detection of the SARS-CoV-2 RNA in human clinical specimens [10]. It was subsequently used for detection of the SARS-CoV-2 virus RNA in veterinary clinical specimens. Similarly, a SARS-CoV-2 surrogate virus neutralization test (sVNT) for the detection of antibodies in human, canine, cat and hamster sera was developed and validated [11]. Since SARS-CoV-2 has been detected in pet cats [1] and since cats are often in close contact with humans, conjunctival, pharyngeal, and serum samples were obtained from cats that were housed in an animal shelter located in a large metropolitan area in Arizona. These samples were tested using the rRT-PCR method [10] and a virus neutralization test kit [11] to determine SARS-CoV-2 infections within the shelter population.

## Materials and methods

### Ethics statement

The study protocol was reviewed and approved by the Midwestern University-Glendale Institutional Animal Care and Use Committee (IACUC # 2997).

### Animal care and sample collection

An animal shelter in Phoenix, Arizona, USA was enrolled in this study. The pet cats were cared for by the shelter staff and attending veterinarians following their standard procedures. The cats were surrendered to the shelter by their former owners or brought to the shelter as strays from the community. Cats were chosen because of their close contact with owners and reportedly their susceptibility to SARS-CoV-2. Cats (n = 350) were randomly selected, and conjunctival and pharyngeal samples were collected from them between July and December 2020. Each swab was placed in 5 ml of sterile phosphate buffered saline (PBS) immediately after collection, vortexed and stored at -80°C until use. The 5 ml volume was necessary to have enough volume for repeat analysis. At the same time, blood samples (1 ml each) from the same 350 cats were also collected, processed and sera stored at -80°C until use. Samples were collected while the cats were anesthetized for an unrelated surgical procedure (spay/neuter surgery). For anesthesia, the cats were given an intramuscular injection of an anesthetic mixture, (DKT) Dexdomitor, ketamine, and butorphanol at a dosage based on the cat’s weight adapted from Plumb’s Veterinary Formulary. Each cat received 20 mcg/kg of the mixture. The cat was then placed on isoflurane (anesthetic gas) and oxygen while the spay, neuter, and sample collection occurred. Immediately following the procedure, cats were given an analgesic Buprenorphine ER (3 mg/ml) at a dose of 0.12 mg/kg subcutaneously.

### Sample size calculation

Sample size was calculated using Scalex and ScalaR calculators [12] to determine the prevalence of SARS-CoV-2 infections in cats. For most prevalence studies a 5% margin of error is considered standard. For an alpha-level of 0.05, expected prevalence of 15%, and 5% margin of error would require at least n = 196 samples. Our final sample size of n = 350 is conservative and sufficient for estimating prevalence with greater precision (approximately 4%) than the widely accepted 5% margin of error.

### Ribonucleic acid (RNA) extraction

RNA was extracted from 400 μl of each saline suspension along with 2 μl of VetMax Xeno RNA internal positive control (IPC) using the MagMax™Viral/Pathogen kit (Applied Biosystems, Waltham, Massachusetts) on a 96-well KingFisher Flex extraction platform with the MVP Flex program, and subsequently eluted in a volume of 50 μl according to the manufacturer’s instructions (Thermo Fisher Scientific).

#### Reverse-Transcriptase Real-Time PCR (rRT-PCR)

The presence of SARS-CoV-2 nucleic acid in the samples was determined by using the Centers for Disease Control and Prevention (CDC) 2019-Novel Coronavirus (2019-nCoV) rRT-PCR emergency use authorization (EUA) protocol (Catalog Number 2019-nCoVEUA-01) that targets the 2019-nCoV nucleocapsid (N) gene (N1 and N2) [10]. Sequences for the primers and probes are shown in Table 1. TaqPath 1-Step RT-qPCR Master Mix (4X) GC was obtained from Thermo Fisher. The 2019-nCoV CDC Probe and Primer Kit for SARS-CoV-2 (KIT-NCOV-PP1-1000) was obtained from LGC Genomics (Beverly, MA). VIC Primer/Probe mix was obtained from Thermo Fisher and used for the Xeno IPC. Amplification reactions were performed in a total volume of 20 μl consisting of 5 μl (1X) TaqPath 1-Step RT-qPCR Master Mix (4X) GC, 1.5 μl of 0.5 μM each primer and 0.125 μM probe, and 5 μl RNA template. Genomic RNA from SARS-CoV-2 (ATCC VR-1986D) was used as the positive control. The negative control was a reaction mixture containing all reagents and 5μl of water in place of RNA template. For the Xeno assay, amplification reactions were as above except that 1 μl of VIC primer/Probe mix was used in place of the N1/N2 primers probe set. The rRT-PCR assay was carried out in an Applied Biosystems 7500 Fast thermal cycler, comprising 2 min at 25°C, 15 min at 50°C, 2 min at 95°C, followed by 45 cycles of 3 sec at 95°C and 30 sec at 55°C. Each sample was tested in duplicate. Occasionally, samples that yielded inconclusive results were sent to the national reference laboratory, United States Department of Agriculture, National Veterinary Services Laboratory (USDA-NVSL), Ames, Iowa for further verification.

**Table 1:** Primers and probes used in this study.

Name	Nucleotide Sequence (5’-3’)	Source
2019-nCoV_N1-F	5’-GAC CCC AAA ATC AGC GAA AT-3’	CDC
2019-nCoV_N1-R	5’-TCT GGT TAC TGC CAG TTG AAT CTG-3’	CDC
2019-nCoV_N1-P	5’-FAM-ACC CCG CAT TAC GTT TGG TGG ACC-BHQ1-3’	CDC
2019-nCoV_N2-F	5’-TTA CAA ACA TTG GCC GCA AA-3’	CDC
2019-nCoV_N2-R	5’-GCG CGA CAT TCC GAA GAA-3’	CDC
2019-nCoV_N2-P	5’-FAM-ACA ATT TGC CCC CAG CGC TTC AG-BHQ1-3’	CDC
N2-CPR	5’-FAM-CGA CTC TTA GGG CTT CTC-BHQ1-3’	This study

### Amplification, cloning, sequencing, and analysis of N2 primers region

Extracted RNA from 8 of 134 samples that yielded inconclusive rRT-PCR results with the N2 primers and probe set and the positive control (ATCC VR-1986D) nucleic acid, were further evaluated by two PCR amplifications. For the first amplification, the reaction mixture was as described above for rRT-PCR, except that N2 forward and reverse primers only (excluding the probe) were used to generate cDNA. The generated cDNA was used in the second round amplification as template in a conventional PCR format that utilized N2 primers only to produce PCR products with compatible ends for cloning into the pCR2.1 vector (Invitrogen). Briefly, amplification reactions were performed in a total volume of 50 μl containing 10 mM Tris-HCL (pH 8.3); 1.5 mM MgCl2; 50 mM KCl; 0.001% gelatin; 200 μM of each deoxynucleoside triphosphate (dATP, dCTP, dGTP, dTTP); 1.0 μM of each N2 primer; 1.25 U of Taq polymerase (Applied Biosystems; Foster City; CA) and 5 μl of cDNA from the first round of PCR as template. The PCR assay was carried out in an Applied Biosystems SimpliAmp thermal cycler, comprising 5 min of pre-incubation at 94°C, followed by 35 cycles of 1 min at 94°C, 1 min at 55°C and 1 min at 72°C. Final extension was performed for 7 min at 72°C. cDNA derived from ATCC VR-1986D was used as the positive control. The negative control was a reaction mixture containing all reagents and 5 μl of molecular grade water in place of DNA template. A portion (10 μl) of the PCR product was separated in a 2% agarose gel following electrophoresis at 70 V for 3 hours using standard methods to check for positive amplification products. Thereafter the remaining PCR products were purified by electroelution and cloned into pCR 2.1 vector following instructions in the Original TA Cloning Kit (Invitrogen, Carlsbad, CA). Positive clones were verified by restriction enzyme analysis and sequenced using universal M13 forward and reverse primers (Functional Biosciences, Madison, Wisconsin). The nucleotide sequences results were analyzed, and multiple sequence alignment and phylogenetic analysis were performed with ClustalW and the neighbor joining method respectively using the MacVector, Inc. Software (Oxford Molecular). Searches for similar sequences in the GenBank database were performed by using the BLAST network service.

### Probe redesign

To verify the reason for the failure of the CDC N2 primers and probe set to produce detectable signals in 41% of the cat samples that were positive for N1 primers and probe set only, a probe designated N2-CPR (Table 1) was designed using an 18-bp conserved sequence within the 60-bp nucleotide sequence results of feline samples. The N2 SARS-CoV-2 sequences differ in this region from those of feline samples by 9-bp substitutions (Fig 2). The primer was synthesized at the Integrated DNA Technologies (Coralville, IA, USA) and validated in our laboratory using a positive SARS-CoV-2 RNA (ATCC VR-1986D), and cat samples that were positive and negative for CDC N2 primers and probe set.

### Surrogate virus neutralization test (sVNT)

SARS-CoV-2 surrogate virus neutralization test kit which was evaluated for the detection of antibodies in human, canine, cat, and hamster sera [11] was obtained from GenScript, Inc., NJ, USA, and used according to the manufacturer’s instructions. Each test sera (n = 350) including the positive and negative controls were diluted 1:10 and 11 μl each was used to perform the assay. Each sample was tested three times.

## Results

### Reverse-Transcriptase Real-Time PCR (rRT-PCR)

All positive and negative controls including Xeno IPC, exhibited expected performance. A sample was considered positive if the cycle threshold (Ct) was ≤40 for both 2019-nCoV markers (N1, N2). If Ct for the sample was <40 for only one of the markers (N1 or N2) the result was inconclusive. A specimen was negative if both N1 and N2 markers had no detectable Ct value. Results are summarized in Table 2. Of the 350 samples tested, 203 (58%) were negative, 145 (41%) were inconclusive, and 2 (0.6%) were positive. The Ct range for the 145 N1 positive samples was 25.41–38.02 with an average of 33.67. The average Ct values for the 2 positive samples were 35.8 and 37.1 respectively for N1 and 39.8 and 39.4 for N2.

**Table 2:** rRT-PCR summary results of pharyngeal, and conjunctival samples.

N1	N2	Ct range	Average Ct	Interpretation	n = 350
not detected	not detected	N/A	0.00	negative	203 (58%)
detected	not detected	25.41–38.02	33.67	Inconclusive	145 (41%)
detected	detected	37.0–40.99	38.99	Positive	2 (0.6%)

### Conventional PCR, gel electrophoresis, and sequencing

**Fig 1:**
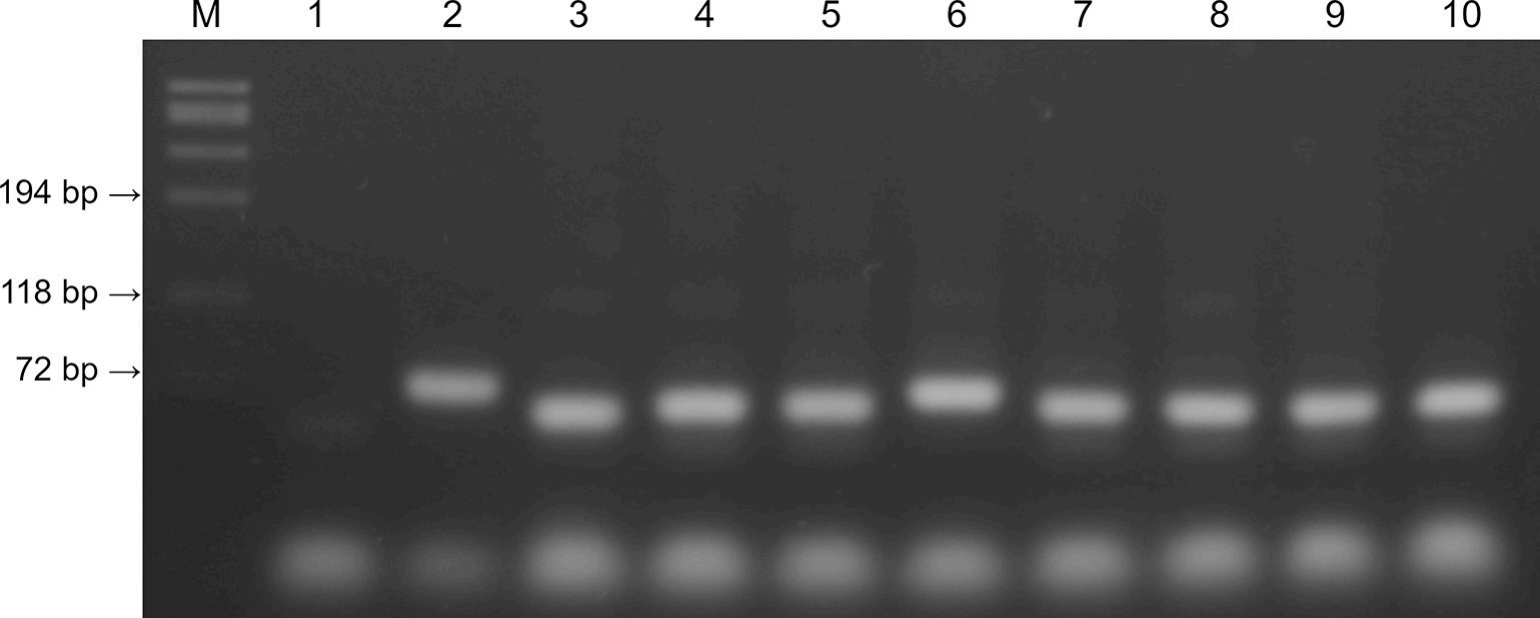
Gel-Red stained agarose gel of PCR products after conventional PCR using the 2019-nCoV_N2-F and 2019-nCoV_N2-R primers (Table 1) and the cDNA generated from first round amplification by rRT-PCR as template. The assay produced the expected 67-bp product of SARS-CoV-2 (lanes 2 and 6) in addition to a second product of 60-bp (lanes 3–5; 7–10) respectively. Lanes: M, *Hae*III-digested øx174 DNA molecular weight standards; 1, negative control (no template); 2; positive control (ATCC VR-1986D); 3–10, feline pharyngeal and conjunctival samples.

**Fig 2:**
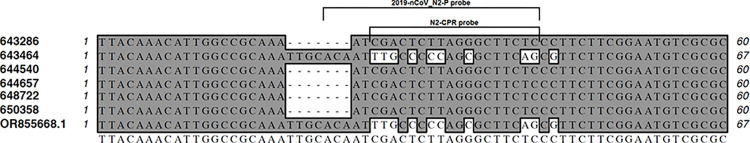
SARS-CoV-2 N2 gene region sequence alignment. Alignment of the nucleotide sequences of the N2 gene region of SARS-CoV-2 from human source (GenBank accession number OR855668.1) with sequences derived from nucleic acid extracts from cat samples. The deletion is boxed and marked with hashes. Base-pair substitutions are boxed. The 2019-nCoV_N2-P and N2-CPR probes are indicated on top of the figure.

**Fig 3:**
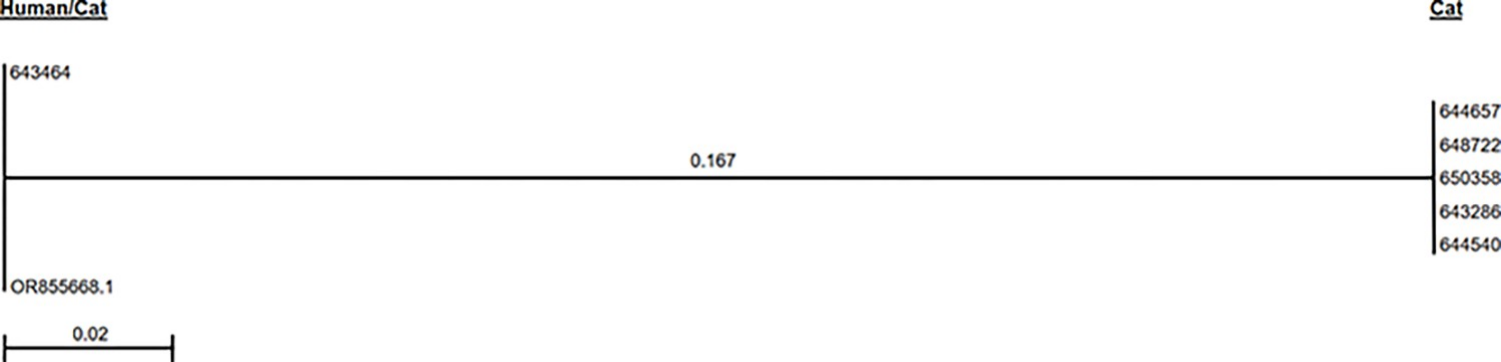
Phylogenetic analysis. Analysis of the nucleotide sequences of the N2 gene region of SARS-CoV-2 and sequences derived from nucleic acid extracts from cat samples using the neighbor joining method showed that the sequences clustered into two distinct groups according to sequence types and are separated from each other with an inter-cluster distance of 0.167. One group consisted of the sequences of SARS-CoV-19 from human at the GenBank database (accession number OR855668.1) and a confirmed positive cat case (643464). The second group consisted of sequences obtained from feline samples RNA extracts consisting of deletion and substitutions compared with the CDC N2 primers probe binding region. The feline sequences were indistinguishable and somewhat related to SARS-CoV-2.

When the cDNA generated from samples that yielded inconclusive results with N2 primer probe set (e.g. one well produced a late positive result and the second well yielded a negative result in a duplicate test) was used in a conventional PCR format, two different fragment sizes were observed. The positive control and a feline sample (Figs 1 and S1, Lanes 2 and 6) produced bands that were slightly larger than the other 7 cat samples. Nucleotide sequencing and analysis of the fragments revealed that the larger bands were 67-bp in length whereas others (lower bands) were 60-bp (Fig 2). Sequence similarity searches using the BLAST network service showed that the nucleotide sequences of the 67-bp fragment was 100% identical to that of human SARS-CoV-2 whereas the 60-bp fragment had 77.61% similarity to that of human SARS-CoV-2 from various geographical locations. Multiple sequence alignment showed that the 60-bp fragment had 7-bp deletion and 10-bp substitutions within the CDC N2 probe binding site, but the N2 forward and reverse primers were conserved (100% identity) (Fig 2). Phylogenetic analysis using the neighbor joining method showed that the sequences clustered into two distinct groups according to sequence types and the groups are separated from each other with an inter-cluster distance of 0.167 (Fig 3). The first group consisted of the sequences of SARS-CoV-19 from human at the GenBank database (accession number OR855668.1) and a confirmed positive cat case (643464). The second group consisted of sequences obtained from cat samples RNA extracts with deletion and substitution within the N2 primers probe binding region. The results indicated that 2 of the cats were infected with SARS-CoV-2 and 123 were possibly infected with virus that is somewhat related to SARS-CoV-2.

### N2-CPR

GenBank database search with the N2-CPR nucleotide sequence did not yield any significant results with sequences at the database. We tested the performance of N2 primers and N2-CPR probe combination (Table 1) against the 123 cat samples that were N2 negative against the CDC N2 primers-probe combination. The samples produced detectable signals with an average Ct of 37.88 (Table 3). This indicated that the negative samples were a result of deletion and base-pair substitutions in the nucleic acid of the unknown agent in the cat samples, and that the N2 gene region of human SARS-CoV-2 is not entirely identical to that same region of the unknown agent. When the N2-CPR was tested against SARS-CoV-2 RNA in a rRT-PCR no amplification was detected indicating lack of cross reactivity.

**Table 3:** CDC N2 primers and N2-CPR probe results for rRT-PCR.

N2-CPR	Ct range	Average Ct	interpretation	N = 127
not detected	N/A	0.00	negative	4
detected	33.33–41.75	37.88	positive	123

### Surrogate virus neutralization test (sVNT)

Percent inhibition values of ≥30% were regarded as positive and values of <30% were regarded as negative [11]. Out of the two cats that had rRT-PCR-confirmed SARS-CoV-2 infection, one was positive (90% inhibition) and the other was negative against the sVNT test, indicating failure of the latter to seroconvert. The remaining samples (n = 348) yielded negative results.

## Discussion

SARS-CoV-2 infections in cats, dogs, and other domestic and wild animals have been reported previously [1]. Because pet cats and dogs are often in close contact with humans, the role of pets in the spread of the virus to their owners and the public are of concern and a One Health issue [13–16]. Continuous monitoring of animals, particularly pets, for the presence of SARS-CoV-2 is important in gathering robust information that may contribute to the development of strategies to reduce exposure incidence. The primary goal of our study was to determine the prevalence of SARS-CoV-2 in cats that were housed in an animal shelter located in a large metropolitan area in Arizona. We used the CDC 2019-Novel Coronavirus (2019-nCoV) rRT-PCR EUA protocol [10], and a sVNT test [11] to determine SARS-CoV-2 infections within the shelter population. Our data indicated that infection rate was low using both rRT-PCR and sVNT, respectively. The low positivity rate agreed with previous studies on 2019-nCoV-2 infections in cats [1,17–19].

It is worth mentioning that the N2 primers and probe combination yielded positive results for both human and feline SARS-CoV-2, with values very close to the cut-off. This indicated that the samples contained a very small amount of the target nucleic acid (low virus load in the samples) as such the PCR reaction would need to run for a high number of cycles to amplify it enough to be detected, resulting in a Ct value close to the cut-off.

An interesting observation was the lack of detectable amplification products with the CDC N2 primers and probe combination (Table 1) against the nucleic acid extracted from cat samples positive with the N1 primers and probe set. Further analysis revealed that the lack of detection was caused by base-pair deletions and substitutions within the primer binding site (Fig 2). This was confirmed by replacing the CDC 2019-nCoV_N2-P with N2-CPR (Table 1). We speculate that cats may harbor a variant of 2019-nCoV-2 or a different virus that may be somewhat related to 2019-nCoV-2. One characteristic of coronaviruses is their high mutation rates including insertions, deletions and genetic recombination which have contributed to diversity allowing infection of numerous animal species [20,21]. It has been reported that mutations (including insertions and deletions) and recombination are two important mechanisms that generate genomic variability in SARS-CoV-2 variants [5]. The nucleocapsid gene is a target for molecular based assays for SARS-CoV-2 and mutations within this gene have been reported by other investigators [22–24] in agreement with our findings. Such mutations have implications as they may affect test diagnostic accuracy and assay failure [25]. Additionally, SARS-CoV-2 variants have been reported based on mutations within the N gene [26,27]. It is therefore likely that a variant not yet described is circulating in cats based on the evidence presented herein. Work is underway to determine the genome sequence of the agent in cats for comparative analysis. Availability of the sequence will shed light into its proper identity and allow for more extensive research. The negative sVNT results on a confirmed rRT-PCR result suggested that the animal did not seroconvert. This observation is in support of previous findings which showed that asymptomatic or mildly symptomatic infections in both humans and animals do not always result in seroconversion [9,28,29]. One can speculate that the level of antibody in the sample at the time of collection may be below the assay detection limit. Alternatively, depending on infection to sampling time the cat may not have had enough time to build antibody against the virus.

A limitation of the study was unavailability of health records of the animals and their owners with respect to 2019-nCoV-2. This information would be valuable in correlating disease transmission from human to animal and from animal to human. The animals did not exhibit obvious clinical signs, and viral shedding may have been missed if the animal cleared the virus prior to specimen collection. Our data is regionally focused, and it may not be true representative of shelters throughout the United States and globally.

In conclusion, our data support previous findings that cats can be infected by 2019-nCoV-2 but at low levels and suggested the possibility of a 2019-nCoV-2 variant or related virus that may be circulating in cat population which in turn may be of unknown risks.

### Nucleotide sequence accession number

The GenBank accession number for the sequence of cat sample 643464 with 100 percent identity to SARS-CoV-2 reported in this paper is PQ730013. Other samples 643286, 644540 644657, 648722, and 650358 are not available in the GenBank but their sequences are reported here in Figs 2 and S2.

## Supporting information

S1 Fig Original gel image used for Fig 1. The image was captured using a UVP MultiDoc-IT gel visualizer (Analytik Jena, Upland, CA). Lane designation is the same as in Fig 1.
(PDF)

S2 Fig Cat sample sequences corresponding to SARS-CoV-2 N2 gene region and derived after PCR (see materials and methods). Samples 643286, 644540, 644657, 648722 and 650358 have deletions and substitutions in comparison with SARS-CoV-2 N2 gene (see Fig 2 for multiple sequence alignment). When BLAST program selection was optimized for somewhat similar sequences (blastn), they yielded 77.61% identity to SARS-CoV-2. Sample 643464 yielded 100% identity to the targeted SARS-CoV-2 N2 gene region. The GenBank accession number is PQ 730013.
(DOCX)
