# Supplementary material for: Detection of SARS-CoV-2 and a possible variant in shelter cats
Source: PLoS One. 2025 Jan 13;20(1):e0317104. doi: 10.1371/journal.pone.0317104 (PMC11730420; doi:10.1371/journal.pone.0317104)
Supplement: S1 Fig — The image was captured using a UVP MultiDoc-IT gel visualizer (Analytik Jena, Upland, CA). Lane designation is the same as in Fig 1. (PDF) [file pone.0317104.s001.pdf]

Raw gel image used for Figure 1

Lanes: X M 1 2 3 4 5 6 7 8 9 10 X X X

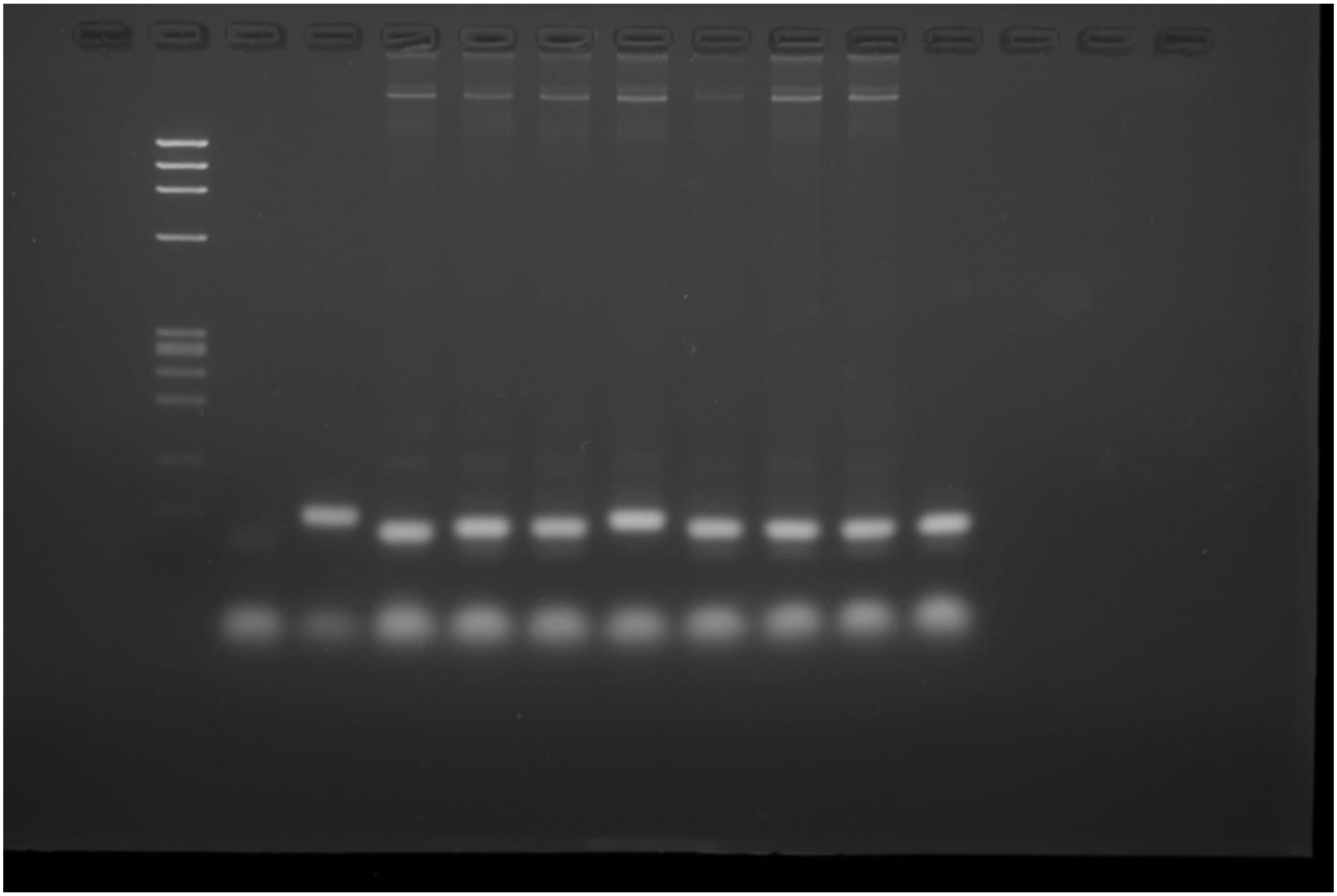

Gel Image was captured using a UVP MultiDoc-It Gel Visualizer (Analytik Jena, Upland, CA).

Loading order:
M: *Hae*III-digested øx174 DNA molecular weight standards
1: negative control (no template)
2: positive control (ATCC VR-1986D)
3-10: Extracted RNA from feline pharyngeal and conjunctival samples following PCR amplification as described under Materials and Methods: Amplification, Cloning, Sequencing, and Analysis of N2 Primers Region
