# Supplementary material for: Detection of SARS-CoV-2 and a possible variant in shelter cats
Source: PLoS One. 2025 Jan 13;20(1):e0317104. doi: 10.1371/journal.pone.0317104 (PMC11730420; doi:10.1371/journal.pone.0317104)
Supplement: S2 Fig — Samples 643286, 644540, 644657, 648722 and 650358 have deletions and substitutions in comparison with SARS-CoV-2 N2 gene (see Fig 2 for multiple sequence alignment). When BLAST program selection was optimized for somewhat similar sequences (blastn), they yielded 77.61% identity to SARS-CoV-2. Sample 643464 yielded 100% identity to the targeted SARS-CoV-2 N2 gene region. The GenBank accession number is PQ 730013. (DOCX) [file pone.0317104.s002.docx]

**643286**

ttacaaacattggccgcaaaatcgactcttagggcttctcccttcttcggaatgtcgcgc

**644540**

ttacaaacattggccgcaaaatcgactcttagggcttctcccttcttcggaatgtcgcgc

**644657**

ttacaaacattggccgcaaaatcgactcttagggcttctcccttcttcggaatgtcgcgc

**648722**

ttacaaacattggccgcaaaatcgactcttagggcttctcccttcttcggaatgtcgcgc

**650358**

ttacaaacattggccgcaaaatcgactcttagggcttctcccttcttcggaatgtcgcgc

**643464** ttacaaacattggccgcaaattgcacaatttgcccccagcgcttcagcgttcttcggaatgtcgcgc

**S2:** Cat sample sequences corresponding to SARS-CoV-2 N2 gene region derived after PCR and sequencing (see materials and methods). Samples 643286, 644540, 644657, 648722 and 650358 have deletions and substitutions in comparison with SARS-CoV-2 N2 gene (see Fig. 2 for multiple sequence alignment). When BLAST program selection was optimized for somewhat similar sequences (blastn), they yielded 77.61 % identity to SARS-CoV-2. Sample 643464 yielded 100% identity to the targeted SARS-CoV-2 N2 gene region. The GenBank accession number is PQ 730013.
